# A Prospective Study of Diet Quality and Mental Health in Adolescents

**DOI:** 10.1371/journal.pone.0024805

**Published:** 2011-09-21

**Authors:** Felice N. Jacka, Peter J. Kremer, Michael Berk, Andrea M. de Silva-Sanigorski, Marjorie Moodie, Eva R. Leslie, Julie A. Pasco, Boyd A. Swinburn

**Affiliations:** 1 Barwon Psychiatric Research Unit, Deakin University, Geelong, Australia; 2 Department of Psychiatry, University of Melbourne, Melbourne, Australia; 3 School of Psychology, Deakin University, Geelong, Australia; 4 Orygen Youth Health, University of Melbourne, Melbourne, Australia; 5 Mental Health Research Institute, Melbourne, Australia; 6 Jack Brockhoff Child Health and Wellbeing Program, Melbourne School of Population Health, University of Melbourne, Melbourne, Australia; 7 Deakin Health Economics, Deakin University, Melbourne, Australia; 8 Barwon Epidemiology and Biostatistics Unit, Deakin University, Geelong, Australia; 9 WHO Collaborating Centre for Obesity Prevention, Deakin University, Geelong, Australia; The University of Queensland, Australia

## Abstract

**Objectives:**

A number of cross-sectional and prospective studies have now been published demonstrating inverse relationships between diet quality and the common mental disorders in adults. However, there are no existing prospective studies of this association in adolescents, the onset period of most disorders, limiting inferences regarding possible causal relationships.

**Methods:**

In this study, 3040 Australian adolescents, aged 11–18 years at baseline, were measured in 2005–6 and 2007–8. Information on diet and mental health was collected by self-report and anthropometric data by trained researchers.

**Results:**

There were cross-sectional, dose response relationships identified between measures of both healthy (positive) and unhealthy (inverse) diets and scores on the emotional subscale of the Pediatric Quality of Life Inventory (PedsQL), where higher scores mean better mental health, before and after adjustments for age, gender, socio-economic status, dieting behaviours, body mass index and physical activity. Higher healthy diet scores at baseline also predicted higher PedsQL scores at follow-up, while higher unhealthy diet scores at baseline predicted lower PedsQL scores at follow-up. Improvements in diet quality were mirrored by improvements in mental health over the follow-up period, while deteriorating diet quality was associated with poorer psychological functioning. Finally, results did not support the reverse causality hypothesis.

**Conclusion:**

This study highlights the importance of diet in adolescence and its potential role in modifying mental health over the life course. Given that the majority of common mental health problems first manifest in adolescence, intervention studies are now required to test the effectiveness of preventing the common mental disorders through dietary modification.

## Introduction

Three quarters of lifetime psychiatric disorders will emerge in adolescence or early adulthood [1]. The National Comorbidity Survey Replication recently reported that more than 22% of adolescents aged 13 to 18 yrs had already experienced a clinically significant mental health problem, with ages of onset ranging from 6 yrs for anxiety disorders, to 13 years for mood disorders [2]. In the last 18 months there have been a number of published studies identifying an inverse associations between diet quality and the common mental disorders, depression and anxiety, in adults [3], [4], [5] and two prospective studies suggesting that diet quality influences the risk for depressive illness in adults over time [6], [7]. While two recent studies have also demonstrated cross-sectional associations between diet quality and emotional and behavioural problems [8] and depression [9] in adolescents, there are no existing studies that examine this association in adolescents prospectively, limiting inferences regarding possible causal relationships. In this study we aimed to investigate relationships between measures of diet quality and adolescent mental health, both cross-sectionally and prospectively. We further aimed to examine the temporal relationships between diet quality and mental health and the associations between change in diet quality and change in psychological symptoms.

## Methods

### Participants

Data for these analyses were derived from the It's Your Move (IYM) project schools in the Barwon-South Western (BSW) region of Victoria, Australia. This region (population 350,109) covers the south-west coast of Victoria and includes the regional centre Geelong (population 205,929 in 2006). The region has 49 secondary schools with a combined enrolment of approximately 49,000. The IYM project aimed to increase the capacity of schools to promote healthy eating and physical activity, increase the awareness of key messages around active transport and healthy nutrition in homes and early childhood settings, and to evaluate the process, impact and outcomes of the project [10].The study design was quasi-experimental, using a longitudinal cohort follow-up. The IYM project involved a sample of 12 schools from the BSW region (eligible students n = 6013). Of these, 3040 adolescents, aged 11 to 18 years, consented to participate at baseline (response rate = 51%). Subsequently, 2054 of these students were followed up (response rate 68%) [11]. Deakin University Human Research Committee approved the ethical aspects of the study and written consent to participate was provided by parents or guardians.

### Questionnaires

Adolescents were sampled in 2005–6 and again in 2007–8. Self-reported information regarding adolescents' key behaviours such as nutrition; mental health and well-being; physical activity; perceptions of the school environment (teachers, canteens, participation in sport); home environment (the role of parents/siblings); neighbourhood environment; and other perception and attitudinal questions was captured with an 84-question survey using Personal Diary Assistants. Surveys were completed during normal class time and took ∼30 minutes to complete. Weight and height were measured by trained researchers.

### Exposure variables

An ordinal Healthy diet score was constructed from the available dietary data. This scoring method was based on those previously developed and validated in adults [12], wherein a point is allotted for each dietary practice in accordance with national healthy eating guidelines. In this study a point was allotted for each healthy dietary practice, based on the recognition of fruit and vegetables as core food groups for previously established dietary scores, including the Alternative Healthy Eating Index and the Mediterranean Index [13] and that diet quality is negatively associated with consuming/purchasing meals outside the home [14]. As such, a point was allotted for each of the following: eating breakfast at home on school days; eating lunch brought from home; consuming two or more fruit serves per day; four or more vegetable serves per day; fruit and/or sandwiches as after school snacks; generally avoiding biscuits, potato chips, pies, hot chips, fried foods, chocolate, sweets, ice-creams as after school snacks; and, finally, both consuming healthy after school snacks and avoiding unhealthy after school snacks. Thus possible scores ranged from 0–7, which was recoded as 1–8. This score was subsequently categorised as 1,2,3 = 1 (“low”, n = 690); 4, 5 = 2 (“medium”, n = 1716); and 6,7,8 = 3 (“high”, n = 508) to aid in reporting and the identification of non-linear relationships. In order to test the sensitivity of this construct, we also examined the summed scores of fruit and vegetable intake per day (for each: one or less = 1; two to three = 2; four or more = 3) as the predictor variable in all analyses and found that it demonstrated an almost identical relationship to the outcomes under examination. As the original scoring method had a better distribution and was in accordance with our previously reported construct [9] we utilised the former in all analyses.

An Unhealthy diet score was constructed using the sum of scores on the following variables: Biscuits, potato chips, other snacks after school; Pies, takeaways or fried foods such as French fries after school; Chocolates, lollies, sweets or ice-creams after school (every day or almost every day = 4; most days = 3; some days = 2; hardly ever or never = 1); plus “In the last 5 school days, how many days did you have non-diet soft drinks?” (0 days = 1 to 5 days = 6); “On the last school day, how many glasses or cans of soft drink did you have? (None = 1 to more than 2 litres = 8); “In the last five school days, how many days did you have fruit drinks or cordials? (0 days = 1 to 5 days = 6); “On the last school day, how many glasses of fruit drinks or cordials did you have? (0 = 1 to 9 glasses = 10); “How often do you usually eat food from a takeaway? (Once a month or less = 1 to Most days = 5); and “In the last five school days, how many days did you buy snack food from shop/takeaway after school? (0 days = 1 to 5 days = 6). The possible range was 9–53, which was subsequently categorised into low (1), medium (2) and high (3) levels of unhealthy food intake based on the distribution of the data.

### Outcome variable

The Pediatric Quality of Life Inventory (PedsQL) is a pediatric general health profile instrument, developed by Varni and colleagues and specifically designed for use with adolescents and children [15]. The PedsQL is a validated measure of depressive symptoms in young adolescents, and is used as an assessment measure for children's mental health [16]. The outcome variable was the emotional functioning subscale of the PedsQL. This variable comprised summed scores on the questions “I feel afraid or scared”; “I feel sad”; “I feel angry”; “I have trouble sleeping”; “I worry about what will happen to me” (0 if it is never a problem; 1 if it is almost never a problem; 2 if it is sometimes a problem; 3 if it is often a problem; 4 if it is almost always a problem). These items are reversed scored and linearly transformed to a 0–100 scale, such that higher scores indicate better quality of life. PedsQL scores were normally distributed and z-scored standardised to yield eventual beta coefficients interpretable as standard deviations (β-z).

### Covariates

Potential confounding factors were identified *a priori*. These included age; gender, area-level socioeconomic status; physical activity levels; dieting behaviours; and body mass index (BMI, calculated as h^2^(m)/wt(kg). We used the Socio-Economic Index For Areas (SEIFA) as an area-level indicator of relative socio-economic advantage and disadvantage. For analysis, SEIFA scores were classified into high SES (≥50%) and low SES (<50%) based on the state-wide median. Physical activity (PA) was measured using summed scores on the questions “In the last 5 school days, how many times did you walk/bike to/from school?” (0–11, recoded to 1–12); “In the last 5 school days, what did you mostly do at morning recess?” and “In the last 5 school days, what did you mostly do at lunch?”(1 = mostly just sat down; 2 = mostly stood or walked around; 3 = mostly played active games); and “In the last 5 school days, how many days after school did you play active sports?” (0–5, recoded to 1–6). Thus, scores ranged from 4–24. Dieting behaviours were categorised as yes (“trying to lose weight” or “trying to gain weight”) or no (“trying to stay at current weight” or “not doing anything about my weight”). BMI was included as a continuous variable. Additional variables constructed for analyses were change in diet quality scores; change in PedsQL scores; change in PA; and change in BMI (all = time 2 minus time 1).

Variables included sequentially as potential confounders in the cross-sectional relationship between diet and mental health included age; gender; SEIFA category; dieting behaviours; BMI; and PA. For prospective analyses, examining dietary scores at baseline as predictors of mental health at follow-up, we also adjusted for baseline PedsQL scores. In analyses examining PedsQL scores at baseline as predictors of diet quality at follow-up, diet scores at baseline were also adjusted for. Finally, in analyses regressing change in PedsQL score on change in diet quality, PedsQL scores and diet quality at baseline; and change in PA and change in BMI were also adjusted for.

### Statistical Analyses

Descriptive statistics were computed and differences between categories of diet scores tested using linear regression analyses for continuous and chi-square statistics for categorical data. Differences between follow-up (those measured twice) and non follow-up (those measured only at baseline) were tested with t-tests or chi-square tests. Differences between genders on diet scores and change in diet scores were examined using t-tests. Cross-sectional analyses were carried out on the data collected in 2005. Multivariable linear regression analyses were used to examine the cross-sectional associations between diet quality and PedsQL. Two main exposures were used in separate regression analyses: Healthy diet and Unhealthy diet scores. Potential confounders were added to the models sequentially (gender; age; SEIFA category; dieting behaviour; BMI; and PA). Effect modification by age group (less than 15 yr or > = 15 yr), gender, SEIFA scores (high or low) and ‘condition’ (intervention or control group) was also assessed for both cross-sectional and prospective relationships.

Multivariable linear regression analyses also examined the relationship of diet quality in 2005–6 to PedsQL scores in 2007–8. In these longitudinal analyses, variables sequentially adjusted for included PedsQL scores at baseline, in addition to those previously documented. In order to examine the issue of reverse causality, we also examined PedsQL scores at baseline as a predictor of diet quality at time 2. We used the dietary scores at time two as the outcome variables in linear regression analyses, with PedsQL scores at baseline as predictors. Diet quality at baseline was adjusted for, in addition to other listed variables. In final analyses, the variable accounting for change in PedsQL scores was regressed on change in dietary scores. Variables, including those previously tested, as well as change in BMI; change in PA; and both PedsQL scores and diet quality scores at baseline, were also adjusted for.

Final analyses included both Healthy and Unhealthy diet scores as continuous predictors of PedsQL scores in the same model, to test the independence and relative contributions of these constructs. This was done for both cross-sectional and prospective analyses.

## Results

Of the initial sample of 3040 students at baseline, 2991 had full data for the PedsQL, 2996 had full data for the dietary intakes and 2921 had full data on relevant covariates. The final sample at baseline consisted of 2915 students with full data available for all three sets of variables (56% males). At follow-up, 2038 had full data for the PedsQL and relevant dietary data and 1958 had full data on relevant covariates. The final follow-up sample, with data available on these three sets of variables, totalled 1949 (54% males). Nearly 60% of the baseline study sample were categorised as “high” SES, based on their SEIFA scores. More than half of students were in the higher category of PA and more than 40% reported dieting behaviour. The majority of students were less than 15 years of age (data not shown). Sample characteristics across categories of diet quality scores at baseline are reported in Table 1. Notably, while high PA was associated with higher Healthy diet scores, it was also associated with higher Unhealthy diet scores, possibly reflecting gender differences; boys had significantly higher scores on the Unhealthy diet scale than girls and were also more active (data not shown). Also counter-intuitively, Unhealthy diet scores were inversely associated with BMI and not associated with SEIFA measures. There were no differences between intervention and comparison schools on diet quality scores or change in diet quality scores and none of the examined variables, including ‘condition’, were identified as effect modifiers (data not shown).Students not followed up were more likely to be male, have higher BMI-z scores and be from the comparison group (p<0.001).

**Table 1:** Characteristics of study participants by category of diet quality score.

*Healthy*	*1*	*2*	*3*	*P value*
Age	14.7 (1.4)	14.6 (1.4)	14.5 (1.4)	<0.001
BMI	21.7 (3.8)	21.7 (3.8)	21.9 (3.9)	0.77
Male	54	58	53	0.02
High Seifa (≥50)	55	60	59	0.03
Dieting behaviour	43	40	45	0.17
High PA (≥median)	46	57	63	<0.001

*Unhealthy*	*1*	*2*	*3*	*P value*
Age	14.7 (1.4)	14.6 (1.4)	14.6 (1.4)	0.02
BMI	22.0 (3.8)	21.9 (3.9)	21.4 (3.7)	<0.001
Male	44	58	64	<0.001
High Seifa (≥50)	61	59	56	0.10
Dieting behaviour	43	39	44	0.03
High PA(≥median)	51	54	60	<0.001

Results given as Mean (SD) or percentage in each category.

**Figure 1:**
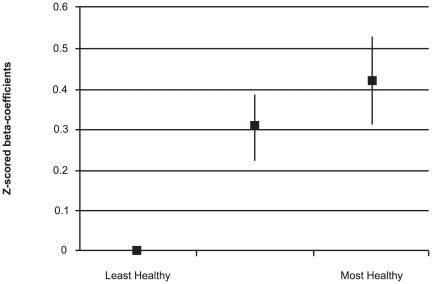
Cross-sectional associations between Healthy diet scores and PedsQL scores (z-score standardized) after adjustments for gender, age, dieting behaviours, BMI, SES and PA.

**Figure 2:**
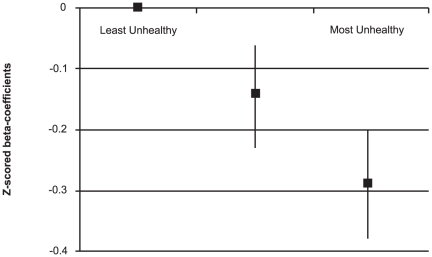
Cross-sectional associations between Unhealthy diet scores and PedsQL scores (z-score standardized) after adjustments for gender, age, dieting behaviours, BMI, SES and PA.

Mean Healthy diet scores were 4.3 (SD±1.7), while mean Unhealthy scores were 20.8 (SD±6.7). Mean Healthy diet scores were similar for males (M = 4.2, SD±6.7) and females (M = 4.3, SD±1.7) (p = 0.27), however mean Unhealthy diet scores were higher for males (M = 21.7, SD±6.7) than females (M = 19.6, SD±6.4) (p<0.001). In cross-sectional analyses, there were positive relationships between Healthy diet scores and PedsQL scores both before and after adjustments (Table 2). These relationships displayed a dose-response pattern (p for linear trend <0.001; Figure 1). There were inverse relationships between Unhealthy diet scores and PedsQL scores before and after adjustments (Table 2) that also demonstrated a dose-response pattern (p for linear trend <0.001; Figure 2). Gender was the only notable confounder in the relationship between Unhealthy diet scores and PedsQL scores, and initial results are reported after adjustment for this variable. Healthy and Unhealthy diet scores were weakly correlated (r = −0.34). When tested as continuous variables in the same model, both were significantly associated with PedsQL scores before and after adjustments, although Healthy diet was a stronger predictor (Healthy: adjusted β-z = 0.14, 95%CI 0.10 to 0.18, p<0.001; Unhealthy: adjusted β-z = −0.09, 95%CI −0.13 to −0.06, p<0.001).

**Table 2:** Cross-sectional associations between diet quality and mental health at baseline: Results of multivariable linear regression analyses.

PedsQL						
	*Crude*	**	**	*Fully adjusted* *		
*Healthy Diet Score*	β-z	*95%CI*	*p-value*	β-z	*95%CI*	*p-value*
Least healthy (n = 690)	0			0		
2 (n = 1716)	0.36	0.27 to 0.45	<0.001	0.31	0.22 to 0.39	<0.001
3 (n = 508)	0.45	0.34 to 0.57	<0.001	0.42	0.31 to 0.53	<0.001

*Unhealthy Diet Score*	*Adjusted Gender*			*Fully adjusted* *		
Least unhealthy (n = 854)	0			0		
2(n = 1165)	−0.13	−0.21 to −0.04	0.001	−0.14	−0.23 to −0.06	0.001
3 (n = 977)	−0.29	−0.38 to −0.20	<0.001	−0.29	−0.38 to −0.20	<0.001

*Adjusted for gender, age, dieting behaviours, BMI, SES and PA.

In longitudinal analyses, Healthy diet scores at baseline predicted PedsQL scores at follow-up, both before and after adjustments for gender, age, SEIFA category, dieting behaviours, BMI, PA and baseline PedsQL scores (Table 3), and again demonstrated a dose-response pattern (p for linear trend <0.001). The association between Unhealthy diet scores at baseline and PedsQL scores at follow-up did not display a dose-response pattern (p for linear trend = 0.28); the highest tertile, but not the second tertile, of Unhealthy diet score was inversely associated with PedsQL scores after adjustments for gender, age, SEIFA category, dieting behaviours, BMI and PA. This association was attenuated after final adjustments for baseline PedsQL scores.

**Table 3:** Longitudinal associations between diet quality at baseline and PedsQL scores at follow-up: Results of multivariable linear regression analyses.

PedsQL									
	*Crude*			*Adjusted* *			+PedsQL baseline		
*Healthy Diet Score*	β-z	*95%CI*	*p-value*	β-z	*95%CI*	*p-value*	β-z	*95%CI*	*p-value*
Least healthy (n = 690)	0			0			0		
2 (n = 1716)	0.26	0.15 to 0.37	<0.001	0.22	0.12 to 0.33	<0.001	0.11	0.01 to 0.21	0.03
3 (n = 508)	0.31	0.17 to 0.45	<0.001	0.29	0.17 to 0.43	<0.001	0.14	0.02 to 0.27	0.03

*Unhealthy Diet Score*	*Adjusted gender*			*Adjusted* *			+*PedsQL baseline*		
Least unhealthy (n = 854)	0			0			0		
2 (n = 1165)	−0.00	−0.11 to 0.10	0.97	−0.01	−0.11 to 0.10	0.82	0.05	−0.04 to 0.15	0.27
3 (n = 977)	−0.15	−0.27 to −0.04	0.01	−0.17	−0.28 to −0.05	0.004	−0.07	−0.18 to 0.03	0.18

*Adjusted for gender, age, dieting behaviours, BMI, SES and PA.

When both diet quality constructs were included as continuous variables in the same model, and the model adjusted for all variables other than mental health at baseline, both were significant predictors of PedsQL scores at follow up (Healthy: adjusted β-z = 0.09, 95%CI 0.04 to 0.14, p<0.001; Unhealthy: adjusted β-z = −0.05, 95%CI −0.10 to −0.001, p = 0.045), but these associations were attenuated by final adjustment for PedsQL scores at baseline (Healthy: adjusted β-z = 0.04, 95%CI −0.01 to 0.08, p = 0.12; Unhealthy: adjusted β-z = −0.02, 95%CI −0.07 to 0.03, p = 0.40).

Over the follow-up period, both Healthy and Unhealthy diet scores decreased. However, the decrease in Healthy diet scores was proportionally greater (mean change = −1.2, SD±2.1; 28% reduction in mean Healthy diet score) than the decrease in Unhealthy diet score (mean change = −1.2, SD±6.2; 6% reduction in mean Unhealthy diet score) indicating that diet quality decreased overall. Males demonstrated a greater decrease in Healthy diet scores over the time period (mean change = −1.3, SD±2.1) compared to females (mean change −0.95, SD±2.2) (p<0.001) and a smaller decrease in Unhealthy diet scores over the follow-up period (mean change −0.77, SD±6.6) compared to females (mean change −1.63, SD±5.6) (p = 0.002).

The hypothesis that change in diet quality would be associated with a change in mental health was supported by the data. Improvements in diet quality, as reflected in increases in Healthy diet scores over the two year follow up period, were associated with increased PedsQL scores (adjusted β-z = 0.21, 95%CI 0.14 to 0.28, p<0.001) over the follow-up period, while increases in Unhealthy diet scores were associated with reductions in PedsQL scores (adjusted β-z = −0.13, 95%CI −0.18 to −0.09, p<0.001), both before and after adjustments for previously described covariates, plus change in PA, change in BMI, dietary scores at baseline and baseline PedsQL scores.

Finally, the hypothesis that mental health at baseline would not predict diet quality at follow-up was supported by the data. After adjustments for gender, age, SEIFA category, dieting behaviours, BMI, PA and diet quality at time 1, PedsQL scores at time 1 did not predict diet scores at time 2 (Healthy diet scores time 2: β-z = −0.01, 95%CI −0.06 to 0.03, p = 0.58; Unhealthy diet scores time 2: β-z = 0.03, 95%CI −0.01 to 0.07, p = 0.12). Age group, gender, SEIFA category and/or condition were not identified as effect modifiers of the relationship between diet quality and PedsQL scores in either cross-sectional or prospective analyses.

## Discussion

In this study, diet quality was associated with adolescent mental health both cross-sectionally and prospectively. Moreover, improvements in diet quality were mirrored by improvements in mental health, while reductions in diet quality were associated with declining psychological functioning over the follow up period. Finally, the reverse causality hypothesis, that the reported associations reflect poorer eating habits as a consequence of mental health problems, was not supported by the available data.

### Study characteristics

These findings from the IYM study are concordant with our previous findings of cross-sectional, dose-response relationships between measures of diet quality and symptomatic depression in Australian adolescents participating in the Healthy Neighbourhoods Study [9]. They extend these previous findings by identifying prospective relationships, by reporting an association between change in diet quality and change in mental health status, and by clarifying the direction of the relationships. However, there are limitations to this study and a range of alternative explanations for our findings should be acknowledged. First, as with all observational studies, we may not have adequately controlled for variables that may act as confounders in the relationship between diet and mental health in adolescents, such as socioeconomic status (SES) or other lifestyle behaviours. However, other studies in adolescents [8], [9] and adults [3], [5], [6], [7] have adjusted for lifestyle behaviours and SES in a multitude of ways and have consistently identified relationships between diet quality and mental health that are independent of these measures. As such, we believe that residual confounding by SES and/or lifestyle is not a likely explanation for the identified relationships.

Another potential explanation is that of unrecognised confounding. In this study we lacked data on familial factors that may promote both poor dietary behaviours and mental health problems in adolescents. Our previous study in Australian adolescents included measures of family conflict and poor family management, as well as dieting behaviours, and did not identify these variables as major confounders in the relationships of interest [9]. Similarly, Oddy et al. [8] identified relationships between measures of diet quality and behavioural problems in adolescents that were independent of family structure and functioning. However, the measures used previously may not have adequately captured the salient aspects of family environment that explain these associations. Data on other potentially important variables, such as personality factors and parental mental health, were also not available.

It is also the case that the study sample was not necessarily representative of the wider Australian population, being drawn from a population with less cultural diversity [11]. This may limit the generalisability of the findings. There may have also been differences between those who chose to participate in the study and those who did not; those who did not may have had worse, or better, mental health. Moreover, there were some differences between those students followed-up and those not followed-up. Those not followed-up were heavier, more likely to be male and in the comparison group. However, as males tended to have higher scores on the Unhealthy diet scale and exhibited more unhealthy changes in their diet over the follow-up period; these differences may have reduced the apparent strength of the association between diet quality and mental health over time.

Finally, there were limitations to the data available to construct the dietary scores. We lacked specific information regarding the composition of meals either brought from or consumed at home and it may be that assumptions regarding the quality of these meals were erroneous. However, previous research has shown that diet quality is negatively associated with consuming/purchasing meals outside the home [14]. The Healthy diet scale also lacked information regarding the consumption of many other components of a healthy diet, such as wholegrains, legumes, lean meats, olive oil, and fish, while the unhealthy diet questions may not have captured all aspects of unhealthy food consumption, such as white bread, fatty and processed meats. However, given that more detailed dietary questionnaires may be unreliable in adolescents [17], simple dietary questionnaires afford sufficient information to rank individuals in terms of their diet quality and we have previously used them successfully to assess the association between diet quality and adolescent depression [9]. Moreover, the intake of core nutrient-dense food groups, fruit and vegetables, yielded almost identical results when compiled as a composite variable in sensitivity analyses, lending weight to the veracity of our measure of healthy diet. Development of a brief, validated measure of diet quality to be used in studies of adolescents would be a valuable contribution to this field of research; however, to our knowledge, no such measurement tool exists at this time.

### Interpretation

Data from cohort studies in the UK suggest that the prevalence of emotional and conduct problems in adolescents increased in the period between the mid 1970's and 1999 [18], [19], while a new meta-analysis, reporting on data collected at many time points and, thus, free of confounding by age and/or recall bias, has reported large generational increases in self-reported psychopathology in American high school and college students between the 1930s and 2007 [20]. These increases did not appear to be explained by social response biases, economic cycles or changes in student populations, and the authors concluded that changes in unidentified cultural factors have resulted in increased rates of psychopathology among American youth.

Paralleling this possible increase in the rates of psychological illness among young people are data indicating a reduction in the quality of adolescents' diets over recent decades. A report based on trends in adolescent food consumption in the US identified a reduction in the consumption of raw fruits, high-nutrient vegetables and dairy foods, which are important sources of fibre and essential nutrients, between 1965 and 1996 [21], with an associated increase in the consumption of fast food, snacks and sweetened beverages [22]. Concurrently, population surveys demonstrate a substantial increase in overweight and obesity among children and adolescents over recent decades [23]. Obesity does not necessarily indicate nutritional repletion, as high-energy foods typically have poor nutrient content [24].

There are many pathways by which an insufficiency of healthful foods and/or an excessive intake of unhealthy and processed foods could increase the risk for mental health problems in adolescents. Fruits and vegetables, as well as other components of a healthy diet such as wholegrains, fish, lean red meats and olive oils, are rich in important nutrients such as folate, magnesium, b-group vitamins, selenium, zinc, mono- and polyunsaturated fatty acids, polyphenols and fibre. Many of these nutrients have already been reported as of relevance in depressive illnesses (e.g. [25], [26]), however the critical importance of these food components as modulators of redox status and immune system functioning, both pathophysiological substrates of depressive illness [27], [28] is increasingly appreciated.

While psychological stress is known to increase the production of pro-inflammatory cytokines, the relationship appears to be bi-directional, with inflammation suggested as a direct contributor to the risk for depressive illness [27]. Inflammation is accompanied by an accumulation of highly reactive oxygen species, and increased oxidative stress is also implicated as a factor in depressive illnesses [28]. Consumption of a diet rich in antioxidants, vitamins, minerals and fibre is associated with reduced systemic inflammation [29]. Conversely, diets that are low in essential nutrients, such as magnesium [30] and western type dietary patterns [31] are associated with increased systemic inflammation.

An important aspect of the shift in habitual diets globally is that of an increase in refined carbohydrate consumption. Hyperglycemia promotes an inflammatory state and high glycemic load (GL) diets are also associated with increased systemic inflammation [32]. It is important to note that low grade systemic inflammation is becoming increasingly common in children and adolescents, as a function of the increasing prevalence of obesity and the metabolic syndrome in this age group. Finally, dietary factors, such as refined sugars and saturated fats, have a detrimental impact on the expression of neurotrophic factors [33] that are particularly salient to depressive illness. Thus, it is plausible to speculate that, by modulating inflammatory, oxidative and/or neurotrophic factors, diet quality influences the genesis and/or progression of depressive illnesses. These hypotheses remain to be tested.

This study highlights the importance of diet in adolescence and its potential role in modifying mental health over the life course. Given that adequate nutrition is essential during periods of rapid physical development, and that the majority of mental health problems first manifest in adolescence and early adulthood, intervention studies are now urgently required to test the effectiveness of preventing the common mental disorders through dietary modification. Moreover, the foods available and provided to adolescents need to be receiving much greater attention. Given the findings from this study, particular attention should now be paid to creating environments that promote healthy eating and engaging parents in supporting adolescents to maintain good nutrition during a difficult life stage.
